# Risk of chronic kidney disease in patients with kidney stones—a nationwide cohort study

**DOI:** 10.1186/s12882-020-01950-2

**Published:** 2020-07-22

**Authors:** Tzung-Fang Chuang, Hung-Chang Hung, Shu-Fen Li, Mei-Wen Lee, Jar-Yuan Pai, Chin-Tun Hung

**Affiliations:** 1 Institute of Medicine, Chung Shan Medical University, Taichung, Taiwan; 2 Department of Internal Medicine, Nantou Hospital, Ministry of Health and Welfare, Nantou, Taiwan; 3 Central Taiwan University of Science and Technology; Department of Healthcare Administration, No. 666, Buzi Rd., Beitun Dist, Taichung City, 406 Taiwan

**Keywords:** Chronic kidney disease, Kidney stones, National Health Insurance database

## Abstract

**Background:**

Chronic kidney disease (CKD) and kidney stones are common in Taiwan; in particular, CKD has a high prevalence but low self-awareness rate. CKD-related risk factors such as diabetes, hypertension, and nephrotoxic drugs are well-known and uncontested; however, kidney stones are relatively less studied and easily overlooked as a risk factor. The objective of this study was to investigate whether kidney stones are a risk factor for CKD.

**Methods:**

We conducted a nationwide population-based matched cohort study to assess the risk of incident CKD in people with kidney stones. Data on incident stones formers in the year 2001—excluding those with a history of CKD—were obtained from Taiwan’s National Health Insurance database. Stone formers were matched (1:4) to control subjects according to sex, age, and index date. The total observation period of the study was 10 years, and the primary end-point was the occurrence of CKD. Student’s t-test and Chi-squared test were used to compare continuous and categorical data, respectively. Logistic regression was used to calculate the odds ratio of kidney stone patients with incident CKD relative to the control group. Cox proportional hazard regression model was used to obtain the hazard ratio for development of incident CKD among patients with kidney stones.

**Results:**

The incidence of CKD in the kidney stone cohort was 11.2%, which was significantly higher than that of the control group (*P* < .001). Survival analysis showed that the stones cohort was 1.82 times more likely to experience CKD than the controls. Age, sex, hypertension, diabetes mellitus, and hyperlipidemia increased the risk of CKD incidence (1.04, 1.27, 1.55, 3.31, and 1.25 times, respectively).

**Conclusion:**

Kidney stones are a definite risk factor for CKD; therefore, patients with stones are suggested to undergo regular renal function monitoring and receive appropriate treatment to avoid CKD.

## Background

The prevalence of urinary tract stones differs by region worldwide and ranges from 5 to 10%. Recent data shows that kidney stones affect approximately 1 in 11 people in the United States [1]. According to Huang et al., the prevalence rate of upper urinary tract stones in Taiwan is as high as 7.38%, with predilection higher in males than in females; furthermore, the recurrence rates were higher in males than in females in their study [2].

The prevalence of chronic kidney disease (CKD) is increasing in developed countries such as the United States of America and Japan. Taiwan also has a high incidence and prevalence of CKD, and Taiwan has the highest prevalence of end-stage renal disease (ESRD) in the world [3]. Wen et al. reported that the national prevalence of CKD in Taiwan was 11.93%, but the rate of awareness of the disease among the patients in their study (462,293 individuals aged older than 20 years) was only 3.54% [4].

Kidney stone is not a major contributor to ESRD. Jungers P et al. reported the overall proportion of nephrolithiasis-related ESRD was only 3.2% [5]. According to the United States Renal Data System 2011 Annual Data Report, only 2% of ESRD were caused by urologic disease which included kidney stones [6]. In other words, kidney stones accounted for few cases of ESRD (< 2%) compared with other major contributors such as diabetes, glomerulonephritis, and hypertension. With such a relatively low proportion of contribution by kidney stones, there might be a tendency to underestimate or ignore the influence of kidney stones on CKD.

Intuitively, one might expect kidney stones to be a risk factor for CKD. Symptomatic stones result in pain secondary to urinary tract obstruction and hematuria due to irritation by the stones. Frequent exposure to nephrotoxic analgesics are a well-known risk factor for CKD. Kidney stones also share many common risk factors with CKD such as low water intake, high protein diet [7], bacterial infection [8], and urinary tract anomalies [9, 10]. In addition, examination using contrast medium [11] and the use of therapeutic methods [12] or even surgical procedures on stone-forming patients might prove harmful to kidney function.

The presence of kidney stones is an infrequent cause of renal failure; however, their occurrence secondary to several kinds of rare hereditary disorders, such as Dent disease, primary hyperoxaluria, 2–8-hydroxyadenine crystalluria, and cystinuria, has been associated with progressive loss of renal function and ESRD at a young age [13–16]. In addition, struvite (infection stones), which form due to repeated bacterial infection, can lead to obstructive nephropathy through the formation of staghorn stones and accounts for the majority of ESRD cases caused by nephrolithiasis [5].

However, most cases of kidney stones are not hereditary. They may occur secondary to factors such as climate, water, dietary habits, living environment, and occupation. The causes for the development of kidney stones in these cases vary, and are infrequently associated with end-stage renal disease. Moreover, local and small-scale studies have found that kidney stones may be a contributing factor in a small percentage of patients with end-stage renal failure and that they may play an influential role in the development of CKD [17]. A case–control study reported a significantly higher prevalence of pre-ESRD kidney stones in African-American hemodialysis patients compared with the general African-American population [18]. Another cross-sectional study revealed decreased creatinine clearance in stone formers compared with normal individuals [19]. However, strong evidence of decreased renal function secondary to kidney stones was not observed in either of the aforementioned studies. A population-based cohort study based in Olmsted County, Minnesota, found that stone formers were at increased risk for a clinical diagnosis of CKD, a sustained elevated serum creatinine level, and a sustained reduced glomerular filtration rate (GFR); therefore, the authors concluded that kidney stones are a risk factor for CKD [20]. The aforementioned study provided strong evidence for the causal relationship between kidney stones and CKD, but the sample was limited to a local county with a small population.

Thus, the present retrospective, nationwide, population-based study was conducted to assess the risk of incident CKD in people with kidney stones in a general population.

## Methods

### Data source

This study comprises a secondary data analysis drawn from the National Health Insurance (NHI) database at the National Health Research Institute of Taiwan. Taiwan’s NHI program, implemented in 1995, is a mandatory single-paid healthcare social insurance covering more than 99% of the population of the country. The NHI Research Database (NHIRD) was commissioned by the NHI Administration and is issued by the National Institutes of Health. It is an epidemiologic research database that provides linked data, including the enrollees’ demographic and registration data as well as claims data from hospitals and general practices (including pharmacy dispensing claims from hospitals, general practices, and community pharmacies). The database provides encrypted information enforced to protect the privacy of the enrollees. Data was collected from the registration files of the medical institutions and the original claims data for reimbursement. The International Classification of Diseases Ninth Revision Clinical Modification (ICD-9-CM) was used to record diagnoses in the NHIRD.

For this study, we used the Longitudinal Health Insurance Database 2000 data set, which contains longitudinally linked data from 1000,000 enrollees (approximately 5% of Taiwan’s population) randomly sampled from the 2000 Registry for Beneficiaries of the NHIRD.

### Study population

**Fig. 1:**
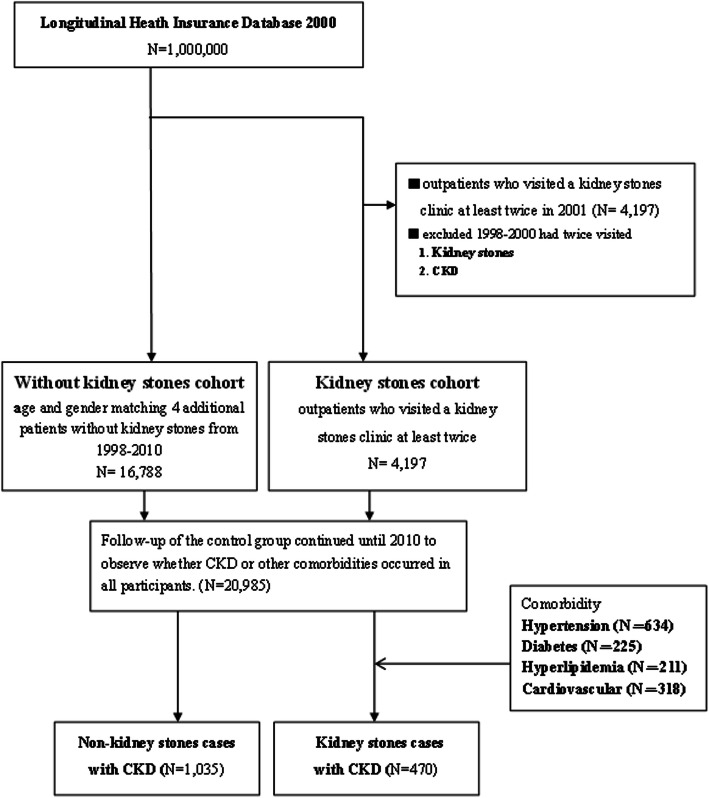
Sampling Framework

The selection of the study population is illustrated in Fig. 1. Patients with kidney stones were selected from this database by identifying the ICD-9-CM diagnostic codes associated with kidney stones (592, 592.0, 592.1, 592.2, 592.9, 594, and 594.0) and other urinary calculi (594.1, 594.2, 594.8, 594.9, and 274.11) in 2001. Outpatients with at least two visits for kidney stones are included in the stone cohort. Patients who had been diagnosed with kidney stones or CKD-related diagnoses (codes 250.4*, 274.1*, 283.11, 403.*1, 404.*2, 404.*3, 440.1, 442.1, 447.3, 572.4, 580–588, 642.1*, and 646.2*) during 1998–2000 were excluded to ensure that only those with incident stone formation in 2001 were included in the study. The stones cohort was observed for the following decade (2001–2010) to identify the occurrence of CKD.

The control cohort was selected from the remaining patients in the database who presented with no history of both CKD and kidney stones between 1998 and 2010. The stones cohort was matched 1:4 to the control cohort according to sex, age, and index date. The total observation period was 10 years, and the primary end-point was the occurrence of CKD.

### Potential confounders

For all individuals in both cohorts, data for potential confounders that are documented as risk factors for CKD, including hypertension (ICD-9-CM codes 402–405), diabetes mellitus (ICD-9-CM codes 250.xx), hyperlipidemia (ICD-9-CM codes 272.xx), and cardiovascular disease (ICD- CM 410–414, 430–438), were obtained. The analysis period was between 1998 and 2000 with at least three appearances of the ICD-9-CM codes for comorbidities.

### Statistical analysis

Analyses were performed using the IBM SPSS statistics version 17.0. All statistical tests were two-sided. *P* < .05 was considered statistically significant. Student’s t-test and Chi-squared test were used to compare continuous and categorical data, respectively. Logistic regression was used to analyze the odds ratio (OR) of CKD patients with urinary tract stones compared with the control group. Binary logistic regression was used to calculate the OR of kidney stone patients with incident CKD compared with the control group. The Cox proportional hazard regression model was used to obtain the hazard ratios (HRs) for development of incident CKD among patients with kidney stones.

## Results

The demographics and potential confounders of the study population are illustrated in Table 1. A total of 4197 kidney stone patients were identified using the following criterion: at least two appearances of the kidney stones–related ICD-9-CM diagnostic codes. The number of males (2762; 65.8%) was twice the number of females (1435; 34.2%) in this study. The average age of the kidney stones cohort was 46.51 ± 14.69 years.

**Table 1:** Demographics and Comorbidities of the Kidney Stones Cohort and the Control Cohort

Variables	Stones cohort (*n*, %) (*N* = 4197)	Control cohort (*n*, %) (*N* = 16,788)	*p*-value
Gender			
Male	2762	11,048	
Female	1435	5740	
Mean age	46.51 ± 14.69	46.51 ± 14.69	
Age stratified			
≤ 30	573 (13.7)	2292 (13.7)	
31–45	1537 (36.6)	6152 (36.6)	
46–60	1292 (30.8)	5168 (30.8)	
> 60	794 (18.9)	3176 (18.9)	
Hypertension			
Yes	634 (15.1)	1890 (11.3)	<.001
No	3563 (84.9)	14,898 (88.7)	
Diabetes			
Yes	225 (5.4)	802 (4.8)	.117
No	3972 (94.6)	15,986(95.2)	
Hyperlipidemia			
Yes	211 (5)	623 (3.7)	<.001
No	3986 (95)	16,165 (96.3)	
CVD			
Yes	318 (7.6)	995 (5.9)	<.001
No	3879(3879)	15,793 (94.1)	

Chi-square test (X [2])

*CVD* Cardiovascular disease

Matching of stones cohort at a ratio of 1:4 according to sex, age, and index date resulted in the identification of 16,788 cases without kidney stones. Cases with kidney stones or a history of CKD over the previous three years (1998–2000) were excluded.

The observation period for the incidence of CKD was 10 years (2001 to 2010). CKD was confirmed by the appearance of CKD-related ICD-9 diagnostic codes (at least twice) during the observation period. Thus, 1505 cases were diagnosed with CKD, accounting for 7.2% of the stones cohort.

Chi-square test results revealed a significant difference in the incidence of CKD between the stones cohort and the controls (Table 2).

**Table 2:** Differences in Chronic Kidney Disease (CKD) Incidence between the Stone Cohort and the Controls

Variable	Stones cohort (*n* = 4197)	Control (*n* = 16,788)	*p*-value
CKD			
Yes	470	1035	<.001
No	3727	15,753	

Chi-square test (X [2])

**Fig. 2:**
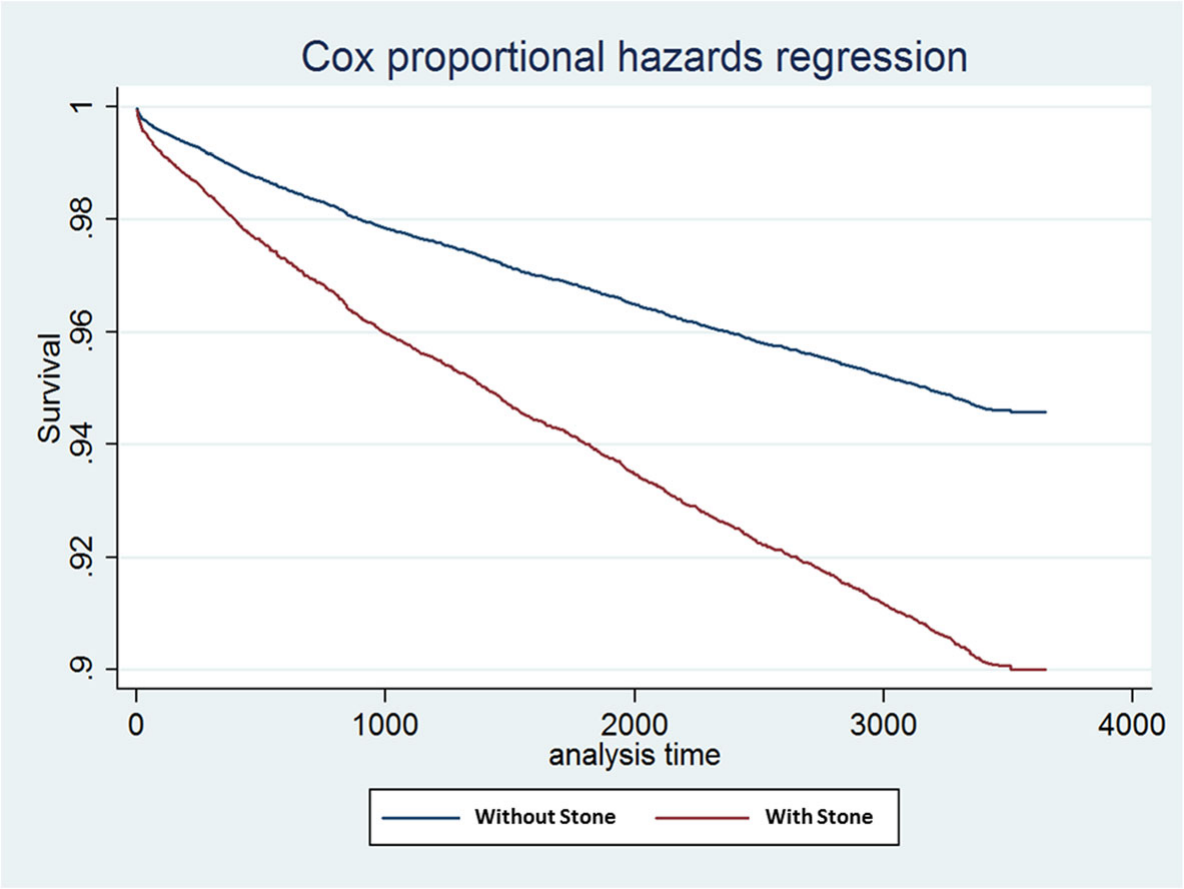
Survival Curves of Chronic Kidney Disease for the Kidney Stones Cohort and Controls (*P* < .001)

Binary logistic regression showed that the stone formers had a higher risk of CKD (OR, 1.94; *P* < .001; Table 3) after adjustment for potential confounders, including age, gender, comorbidities (hypertension, diabetes, hyperlipidemia, and cardiovascular disease). Cox proportional hazard regression models adjusted for age, gender, and comorbidities used to assess the risk for incident CKD in the stone formers revealed a HR of 1.82 (*P* < .001; Table 4). The survival curves of CKD-free probabilities for patients with or without kidney stones are illustrated in Fig. 2; CKD-free probability in the kidney stones cohort was significantly lower than that in the non–kidney stones controls (*P* < .01).

**Table 3:** Odds Ratio (OR) of Chronic Kidney Disease for the Various Predictors

Variable	OR	OR, 95% CI	*p*-value
Age	1.03	1.03–1.04	<.001
Gender (Male)	1.25	1.11–1.41	<.001
Stones or not	1.94	1.72–2.19	<.001
Hypertension	1.64	1.42–1.90	<.001
Diabetes	3.75	3.19–4.40	<.001
Hyperlipidemia	1.37	1.12–1.67	.002
CVD	0.97	0.81–1.16	.742

Binary logistic regression

*OR* Odds ratio, *CVD* Cardiovascular disease, *CI* Confidence interval

**Table 4:** Hazard Ratio of Chronic Kidney Disease for Various Predictors Obtained Using the Cox Proportional Hazard Model

	HR	HR, 95% CI	*p*-value
Age	1.04	1.03–1.04	<.001
Gender (Male)	1.27	1.14–1.41	<.001
Stones or not	1.82	1.63–2.02	<.001
Hypertension	1.55	1.36–1.77	<.001
Diabetes	3.31	2.87–3.80	<.001
Hyperlipidemia	1.25	1.05–1.48	.013
CVD	1.04	0.89–1.22	.613

*HR* Hazard ratio, *CVD* Cardiovascular disease

## Discussion

After adjusting for potential confounders, we found that patients with kidney stones were 1.82 times more likely to develop CKD during the 10-year follow-up period (HR = 1.82; 95% CI 1.63–2.02) compared with the general population. To the best of our knowledge, this is the first nationwide population-based cohort study to investigate the risk of CKD in patients with kidney stones.

This study focused on acquired or environmental associated kidney stones but not hereditary related cases. To confirm that the stone cohort comprised patients with acquired stones, cases presenting with a history of kidney stones for the past three years (1998–2000) were excluded. Therefore, the exposure times between the cases in the stone cohort were similar. The inclusion criterion for the incident stone cases was the appearance of ICD-9-CM diagnosis codes of kidney stone (a minimum of two times because a single appearance of the code may prove as a false positive).

CKD development requires a long period of exposure to the risk factors; therefore, we observed the patients for a period of 10 years [21]. As with kidney stones, the identification of CKD also relied on the appearance (at least two times) of the ICD-9-CM diagnosis codes associated with CKD.

Potential confounders were identified under conditions that were more stringent: at least three appearance of the ICD-9-CM codes for CKD-related comorbidities. In addition to diabetes, hypertension, hyperlipidemia, and cardiovascular diseases were more common in the stone cohort than in the controls (Table 1).

Kidney stones are more prevalent in middle-aged males, with a male to female ratio of approximately 2:1. The incidence of CKD in patients with kidney stones was approximately 11.2%, which was a crude estimation based on 470 patients out of the 4197 experiencing CKD in the kidney stones cohort group.

Kidney stones are the predictors and risk factors of CKD, and their importance in the present study can be noted by their high prevalence rates (5–10%), OR (1.94), and HR (1.82), all of which were second to only those of diabetes (Tables 3 and 4).

The strength of the current study is that it is a nationwide population-based cohort study that could demonstrate the causal relationship between kidney stones and CKD. Because of the mandatory insurance policy of the NHI, the integrity and representativeness of the information is thorough compared with other regional research surveys. Moreover, Taiwan’s NHI has been in existence for more than two decades (since 1995) [22, 23], providing a sufficient time period to make observations; the rate of data loss is very low, unless the insurant has emigrated to another country or the insurance is suspended because of other special reasons. Zero values were found to be missing during the sampling of this study. Therefore, our data, which covers a national source of information, are highly representative and can be used as reference for developing epidemiology, public health research, health care policy, and clinical guidelines. Although several local and small-scale studies have explored the relationship between kidney stones and CKD [17–20], a nationwide population-based cohort study has not been published so far.

However, this study has some limitations. First, the NHIRD does not provide detailed information on precise relevant clinical variables such as laboratory data and imaging or pathologic findings for the kidney disease. Information about the severity of kidney stones, the composition and types of stones, and staging of CKD are also lacking. Second, the level of evidence derived from cohort studies is generally lower than that from randomized controlled trials because of potential biases related to unknown confounders that cannot be adjusted for [24]. Third, data on symptoms related to kidney stones, such as hematuria, colicky pain, obstruction, and infection, were unavailable, as was information on relevant therapies, such as lithotripsy or stone surgery, and medications used during treatment. These factors may have potentially affected the analysis in the current study.

Kidney stones contributed to only a small proportion of ESRD patients receiving hemodialysis [5, 6], and the contribution was much lower than that of diabetes, hypertension, and hyperlipidemia. Given the slow progression of the clinical course of CKD and the minimal signs and symptoms encountered, patients often neglect the presence of this condition. Furthermore, the effect of kidney stones on CKD is probably underestimated by the clinical doctors, government health authorities, health care institutions, and policy decision makers.

## Conclusion

In conclusion, the results of the present study suggest that kidney stones are a clear and independent risk factor for CKD; hence, patients with kidney stones should receive regular kidney function monitoring and obtain appropriate treatment to prevent or delay the development and progression of CKD.

## Data Availability

Data are available from the National Health Insurance Research Database (NHIRD) published by Taiwan National Health Insurance (NHI) Bureau. Due to legal restrictions imposed by the government of Taiwan in relation to the “Personal Information Protection Act”, data cannot be made publicly available. Requests for data can be sent as a formal proposal to the NHIRD (http://nhird.nhri.org.tw).

## Abbreviations

CKD: Chronic kidney disease
ESRD: End stage renal disease
GFR: Glomerular filtration rate
NHI: National Health Insurance
NHIRD: NHI Research Database
ICD-9-CM: International Classification of Diseases Ninth Revision Clinical Modification
OR: Odds ratio
HR: Hazard ratio
